# Flow Cytometric Analyses of Lymphocyte Markers in Immune Oncology: A Comprehensive Guidance for Validation Practice According to Laws and Standards

**DOI:** 10.3389/fimmu.2020.02169

**Published:** 2020-09-17

**Authors:** Claude Lambert, Gulderen Yanikkaya Demirel, Thomas Keller, Frank Preijers, Katherina Psarra, Matthias Schiemann, Mustafa Özçürümez, Ulrich Sack

**Affiliations:** ^1^University Hospital, Immunology Laboratory, FRE-CNRS 3312, Saint-Etienne, France; ^2^Stem Cell Laboratory, Immunology Department, Faculty of Medicine, Yeditepe University, Istanbul, Turkey; ^3^Acomed Statistik, Leipzig, Germany; ^4^Laboratory of Hematology, Department of Laboratory Medicine, Radboud University Medical Center, Nijmegen, Netherlands; ^5^Department of Immunology-Histocompatibility, Evangelismos Hospital, Athens, Greece; ^6^Institute for Medical Microbiology, Immunology and Hygiene, Technische Universität München, Munich, Germany; ^7^Universitätsklinikum Knappschaftskrankenhaus Bochum, Bochum, Germany; ^8^Medizinische Fakultät, Institut für Klinische Immunologie, Universität Leipzig, Leipzig, Germany

**Keywords:** flow cytometry, procedures, accreditation, quality control, laboratory diagnostics, validation

## Abstract

Many anticancer therapies such as antibody-based therapies, cellular therapeutics (e.g., genetically modified cells, regulators of cytokine signaling, and signal transduction), and other biologically tailored interventions strongly influence the immune system and require tools for research, diagnosis, and monitoring. In flow cytometry, *in vitro* diagnostic (IVD) test kits that have been compiled and validated by the manufacturer are not available for all requirements. Laboratories are therefore usually dependent on modifying commercially available assays or, most often, developing them to meet clinical needs. However, both variants must then undergo full validation to fulfill the IVD regulatory requirements. Flow cytometric immunophenotyping is a multiparametric analysis of parameters, some of which have to be repeatedly adjusted; that must be considered when developing specific antibody panels. Careful adjustments of general rules are required to meet legal and regulatory requirements in the analysis of these assays. Here, we describe the relevant regulatory framework for flow cytometry-based assays and describe methods for the introduction of new antibody combinations into routine work including development of performance specifications, validation, and statistical methodology for design and analysis of the experiments. The aim is to increase reliability, efficiency, and auditability after the introduction of in-house-developed flow cytometry assays.

## Introduction

Medical routine and study laboratories are subject to a large number of regulations. Recommendations on standard practices for flow cytometry (FCM) validation procedures must comply with legal obligations, the European Regulation 2017/746 on *in vitro* diagnostic medical devices (EU-IVD-R), which also contains mandatory requirements for *in vitro* diagnostic medical devices (IVD) developed and manufactured in healthcare facilities within the European Union (1).

FCM is applied in different analytical fields that comprise assays for research use only (RUO), preclinical applications (PCA) as well as routine methods provided as medical laboratory services. Quality standards for RUO assays and PCA depend on specific rules set by authorities or research and development (R&D) institution, respectively. A common framework for research reporting is the “Minimum Information about a Flow Cytometry Experiment” (2); preclinical rules depend on the context.

Immune therapies for tumors require manifold flow cytometric support. Firstly, while detection of circulating tumor cells is still experimental (3), diagnosis of leukemias and lymphomas is well-established, and a few IVD test kits already exist. Secondly, monitoring of hematological and solid tumor response to therapy is increasingly important, especially in antibody therapies, e.g., reduction of normal of malignant B cell counts following antibody therapy (4), detection of checkpoint inhibitor receptor expression (5), or quantification of CAR-T cells following CAR-T cell therapy (6). Next, detection of adverse effects of novel therapies on lymphocyte subpopulations and their functions supports best medical practice and provides additional knowledge in novel treatments (7).

Our recommendation aims to provide guidance to fulfill legal and normative obligations of EU-IVD-R and EN ISO 15189 (ISO), respectively. Technical terms given in the following recommendations were taken from International vocabulary of metrology (VIM)—Basic and general concepts and associated terms (8). Technical terms from the EU IVD-R are preferred because of their mandatory character in cases of lack of conformity with VIM.

FCM encompasses a wide range of different methodological approaches. It is not in the scope of this article to provide detailed experimental protocols that consistently cover all FCM-based applications. Rather, our focus is on aspects that (i) address specific problems of FCM for novel diagnostic requests, (ii) are common to most FCM-based assays intended for use as a medical laboratory service, and (iii) are minimal experimental requirements that are mandatory to fulfill the above mentioned legal and normative obligations.

## Legal and Regulatory Obligations

EU-IVD-R defines IVD as “…any medical device which is a reagent, reagent product, calibrator, control material, kit, instrument, apparatus, piece of equipment, software or system, whether used alone or in combination, intended by the manufacturer to be used *in vitro* for the examination of specimens, including blood and tissue donations, derived from the human body…” in the field of medical healthcare (1). The CE (Conformité Européenne) mark certifies that an IVD is in compliance with the European *In vitro* Medical Device Directive 98/79/EC. According to EU-IVD-R, the use of CE-marked IVDs is mandatory for all laboratories that perform diagnostic tests in patient care. So-called in-house tests can only be employed if no product with CE marking is available on the market that meets the appropriate level of performance, which is the case for many parameters in the field of immune oncology. Laboratories must also comply with EN ISO 15189 or, where applicable, appropriate national regulations. Minimum standards are the general safety and performance requirements according to Annex I of the EU IVD-R. Furthermore, a documented risk management system as well as the definition and evaluation of analytical or clinical performance characteristics must be maintained throughout the entire life cycle of an IVD.

**ISO 15189** (9) aims to implement the quality assurance policy into medical laboratory services (10–12). This must consider biological and technical specificities encountered in some technique such as in quantitative cell analysis (cytometry) as recently discussed (13, 14).

There are numerous relationships between the requirements of the EU-IVD-R (1) and ISO 15189 (9), which are further modified by national legislation. ISO 15189 accreditation covers laboratory management and technical issues. The first part addresses general laboratory organization in detail (9). The second part addresses technical issues (Supplement I) classified under Ishikawa (Fishbone) diagram (15). Much information is common to any analysis:

- Operator authorization (ISO 15189 chapter 5.1),
- Environment (5.2),
- Instruments and reagents (5.3),
- Sampling and pre-analytics (5.4),
- Validation, metrology, or contamination (5.5), (5.6),
- Post-analytics and reporting (5.7 to 5.9), and
- Laboratory information management system (LIMS; 5.10) (9).

Additional information is highly specific to each analysis: method settings, validation, exclusion of interferences (5.5), and quality control and standardization (5.6).

ISO 15189 allows a flexible scope that is highly recommended to FCM laboratories. Flexible scope allows continuous expansion of the range of flow cytometric parameters. This depends on well-established validation procedures, followed by continuous evaluation and occasional improvements. This must be periodically supervised by audits, reports, and management reviews (14).

Various aspects of laboratory management (Quality management, LIMS, agreements, client feedback, complaints, etc.) as well as of the analytical process (measurement, “mother nature”) follow general rules of laboratory diagnostics and will not be discussed here. In contrast, manpower, material, machine, and method require serious consideration in the field of FCM for which consensual resolution is needed. Various national activities have been published to support laboratories in the validation process, for instance in Brazil (16) or Germany (17, 18).

## Common Practice in Immune Oncological Flow Cytometry

Whereas, the EU-IVD-R determines the necessary properties to be validated, both general and FCM specific guidelines have been developed that provide more detailed information regarding the experimental design and statistical methods for analysis. In particular, the guidelines developed by the Clinical and Laboratory Standards Institute (CLSI “evaluation protocols”) are quite helpful (19–21). However, adaptation of the guidelines to flow cytometry is challenging.

Several attempts have been made to develop guidance for method validation experiments for flow cytometry-based assays (22, 23). Although the guidance by Selliah et al. (23) provides a wide range of experiments as well as acceptance criteria, the statistical methodology, including the rules for deriving the necessary sample sizes, do not correspond to the state of the art.

Finally, it must be mentioned that there is still inconsistency in the terms used to describe parameters to validate. For example, in the EU-IVD-R the term “analytical sensitivity” is still used although the definition of limits of detection (LoD) and quantitation (LoQ) offer a more precise description of the underlying concepts. Another example is the use of the term “accuracy.” It is differently defined in the pharmaceutical world as describes “the systematical error of a measurement” (24), while in the laboratory medicine community where accuracy encompasses both systematic and random errors. Internationally accepted white papers and protocols have been published on this topic (23, 25). The aim of our paper is to propose a reasonable but also efficient consensus strategy for introducing laboratory-developed panels and performing method validation in clinical FCM laboratories as well as to propose minimal criteria to fulfill.

## What Makes FCM so Unique?

Guidance for method validation in FCM is hard to establish due to the complex nature of this technology. This includes the requirement for samples, the fact that cell characterization requires multiple parameters which can be evaluated in different combination and the high number of interacting variables in each experiment. This will become even more complicated in future when high-parameter research methods such as clustering become routine (26). There are many different clustering algorithms for evaluation of cytometry results. The Flow Cytometry Critical Assessment of Population Identification Methods (Flow-CAP) challenge has made a comparison of performance for flow cytometry clustering algorithms (27). They found that these programs are not accurate enough and too slow for routine use. While specific programs were found to be accurate, slowness rendered them impractical for routine use in clinical laboratories. New algorithms are being developed that address these problems (28).

Relevant parts of the laboratory process are shown in Box 1.

Box 1 **The laboratory process.**

- The pre-analytical phase. Functional assays and some differentiation markers are time- sensitive and require an analysis to be performed within a few hours of blood draw whereas some analyses can still be correctly performed within 72 h. This must be validated for each parameter that is being analyzed.
- In the analytical phase, almost all items to be reported in standard operating procedures (SOP) (including linked documents) are themselves still in need of standardization, including protocol design, international references, operator confirmation, and analytical performances as well as description of the assay principle, validation process, and supervisor authorization.
- The post-analytical phase comprises (i) the technical review of examination results as well as (ii) a plausibility check of the results prior to release. A major issue of post-analytics is to provide valid reference ranges or decision limits.

The major error sources in FCM (Box 2) are related to (i) sample quality, (ii) protocol and panel design, (iii) methods used for instrument settings, standardization, discrimination of negative or positive populations and absolute counting and (iv) data analysis and interpretation (29). Panels must be well-designed and spectral overlap must be sufficiently recognized and properly compensated (30, 31).

Box 2 **Error sources in flow cytometry.**

- Daily instrument variation is at risk and must be measured and minimized as much as possible by tracking instrument and reagent stabilities. For clinical labs, CE-labeled cytometers should be used, and manufacturers' advice must be followed.
- Protocol outlines for sample preparation, fluorophore detection and gating strategy are often ill-defined and lack consensus.
- One analysis simultaneously identifies several cell sub-populations and provides as many results. Unlike in most diagnostic tests, one analysis does not mean one result.
- Phenotype definitions are not univocal and are constantly changing. There is no international “gold standard” for determining accuracy in terms of phenotype or absolute quantitative measurements.
- Some analyses such as leukemia typing, or functional investigation require several assays (protocols) and their interpretation require the integration of information from the multiple assays.
- Specificity of antibodies used for the detection of antigens may vary depending on the clone, conjugate, and manufacturer. In contrast, different clones can recognize the same antigen and can be certified through the Human Leukocyte Antigen determination program (32).
- There are many different typical phenotypes that need to be identified in the diagnosis of all possible diseases. Samples are frequently scarce and include bone marrow, punctates, and other biological fluids in addition to various anticoagulated blood. All these samples must be fresh for analysis. It is therefore not possible to have internal quality control (IQC) for each analysis, sample type, or pathological phenotype. However, a few IQC are commercially available, mainly for CD4+/CD8+ T cells or CD34+ stem cells. These IQC can be stored for weeks thanks to stabilizing treatment. Not all cell types could be investigated, and specific needs for immune oncology are not yet met.
- As a result of the continuously evolving landscape of biological understanding, new therapies and technological capabilities, newly optimized antibody combinations must often be incorporated into FCM assays. It is therefore important that protocols must their flexibility.
- Although samples are prepared and analyzed in parallel and several batches can be analyzed in 1 day, each sample is prepared individually with independent risks of error and variability. The analysis of one test within a batch does not depend on the whole batch as it is for microtiter-based serological immunoassays with one common standard curve. The validation of IQC inside the batch does not full guarantee the quality of each analysis. Inversely, a successful analysis on one sample, including eventually one IQC does not necessarily mean the entire batch is valid.
- For the same reasons, external quality assessment (EQA) schemes are rare (http://www.eptis.org). The majority are only available for a small number of analyses, in preserved (meaning altered) conditions. Schemes providing fresh blood samples are rare and expensive (http://www.instandev.de/en.html).
- In absence of international references, absolute counts (in cell concentration or antigen density as well) slightly differ according to the system used as shown in EQA comparisons (33).
- The risk for contamination between samples is not negligible. Samples in a batch can have extreme concentration of at least one cell subset. The sample-to-sample contamination risk depends on the organization of the sample preparation (proximity of the tubes, changes in tips or probe cleaning, and on the efficacy of the probe washing between two consecutive samples.

## Types of Flow Cytometric Assays

Quantitative analyses allow the quantitation of precisely defined cell subsets, even as absolute values. Some EQA and standardization guidelines are available. They can address rare events with a need for high sensitivity (low LoQ).

Quantitation of very rare events has recently been developed for the assessment of residual disease and requires precautions to obtain good repeatability at high sensitivity. A minimum number of parameters and a minimum number of positive events to be recorded are required, which means that the sensitivity up to 0.01 or even 0.001% of leukocytes can only be achieved if at least 3 × 10^5^ to 3 × 10^6^ events are acquired (34). In Table 1, cell counts to be analyzed when quantifying rare cells are shown.

**Table 1:** Total number of cells to collect in detection of rare events.

**Frequency of Rare Events (1/x)**	**% of total**	**Desired coefficient of variation % (rare events required)**			
		**30 (11)**	**10 (100)**	**5 (400)**	**3 (1,111)**
20	5	222	2,000	8,000	22,222
50	2	556	5,000	20,000	55,556
100	1	1,111	10,000	40,000	111,111
1,000	0.1	11,111	100,000	400,000	1,111,111
10,000	0.01	111,111	1,000,000	4,000,000	11,111,111
100,000	0.001	1,111,111	10,000,000	40,000,000	111,111,111
1,000,000	0.0001	11,111,111	100,000,000	400,000,000	1,111,111,111

*For very rare cell populations, number of cells to be analyzed increases substantially*.

Most of FCM analyses are qualitative in nature. They mainly address the identification of cells, such as the diagnosis of leukemia and lymphoma, immune monitoring, or in proliferative or dysplastic disorders. Partial quantitation (%) is then determined and informative but not clinically critical. Standardization and EQC are frequently not available and IQC are rare. Measurement of precision, accuracy, or working range is not relevant.

Functional analyses usually require challenging fresh samples with different stimulants. In this case, quantitation is important but rarely standardized. Calculation of precision is done by repeating stimulations. The working range can be estimated by testing different concentrations of the stimulant. Sensitivity is estimated by the lower stimulation dose giving a significantly different readout from the negative control. Comparing positive and negative controls offers information of reproducibility of the assays and the frequency of “non-responders” observed for some assays. Measuring accuracy is generally not possible. Inter-laboratory comparison is difficult to organize as samples must be tested within 1 day. Standardization and multi-center clinical evaluations are needed.

## Validation of Flow Cytometric Assays

Based on the specific characteristics of FCM mentioned above, procedures must be adapted to render method validation more efficient but realistic in daily practice. First, analytical and clinical validation must be distinguished. Clinical validation (diagnostic accuracy, e.g., sensitivity and specificity) is commonly based on clinical studies. Patient data are usually not accessible for laboratories. This is not the scope of this paper but is briefly shown in Table 2.

**Table 2:** Clinical performance characteristics given by EU-IVD-R that shall be stated by manufacturers to state “fitness for purpose” need to be maintained during the lifetime of an IVD.

**Term**	**Definition/explanation**	**Comments**	**Specific considerations for flow cytometry**
**CLINICAL PERFORMANCE**			
Diagnostic sensitivity	Test positivity in disease, true positive fraction, ability of a test to correctly identify disease at a particular decision threshold (35). In agreement or concordance studies, where the true disease state is not available but the test result of a reference method, the term “percent positive agreement” (PPA) is used instead of sensitivity.	“Diagnostic sensitivity” is used in Europe and “clinical sensitivity” is used in the United States (36). This also applies to “diagnostic specificity”. The following question is addressed: To what degree does the test reflect the true disease state? The sensitivity is the fraction of patients correctly identified by the test to have the disease (true test positives) among all patients with the disease (as defined by an independent reference standard). Note that the cut-off should be chosen prospectively according the costs of false positive and false negative results. Data driven approaches like choice of the cut-off according maximum Youden-Index is not recommended because of its high uncertainty. The sensitivity does not depend on the prevalence of the disease, but on the spectrum of patients in the disease or non-disease group, respectively.	Clinical performance assessment requires sufficient analytical evaluation. The initial analytical performance assessment must include “abnormal” samples, which must be distinguishable from normal or negative samples, respectively. Crucial for any diagnostic performance study are well defined clinical conditions that specify positivity. Even though clinical performance assessment is mostly done by clinical studies, laboratories are encouraged to retrospectively evaluate the diagnostic sensitivity of their reported results. In such cases, it is crucial to offer the attending physician structured forms that enable him to provide specific clinical information about the patient and the underlying disease or clinical question. Further information necessary for the evaluation of the results should also be requested. Ideally, the reporting of the diagnostic findings is followed by a follow-up communication with the attending physician, if the latter has information that are relevant to the assessment of diagnostic sensitivity. Since neither clinical studies nor retrospectively assessed diagnostic sensitivity may be suitable to some FCM tests, labs are encouraged to thoroughly perform vertical plausibility checks including all available information in case of follow up investigations.
Diagnostic specificity	Test negativity in healthy, true negative fraction, ability of a test to identify the absence of disease at a particular decision (35). In agreement or concordance studies, where the true disease state is not available but the test result of a reference method, the term “percent negative agreement” (NPA) is used instead of specificity.	The following question is addressed: To what degree does the test reflect the true disease state? The specificity (spec) is the fraction of patients correctly identified by the test to not have the disease (true test negatives), among all patients without the disease (as defined by an independent reference standard). The specificity does not depend on the prevalence of the disease, but on the spectrum of patients in the disease or non-disease group, respectively.	As stated for sensitivity, diagnostic specificity assessment also relies on enough initial analytical performance studies. Clinical studies, a retrospective evaluation and thoroughly plausibility checks are proposed that need to be planned and documented with respect to form sheets provided and assessment strategies. Well-designed panels and protocols provide information for the specificity. Documentation for correlation of cytometry results with other laboratory data for the specific clinical diagnosis is necessary.
Positive predictive value	The percentage of positive test results that are true positives when the test is applied to a population containing both healthy and diseased subjects (35). Note: The positive predictive value varies with the prevalence of the disease in the population tested.	The following question is addressed: How likely is the disease given the test results? The positive predictive value (PPV) describes the perspective of a physician or a patient in view of a positive test result: It is the probability that the patient has the disease (as defined by an independent reference standard) given a positive test result or (post-test probability). The PPV depends on the prevalence of the disease. Its value corresponds to the clinical situation where the test is applied. When a test has a PPV > prevalence, it might have a good diagnostic performance (considering a similar consideration for the NPV in parallel).	Immunophenotyping of certain diseases with special markers, provides information on positive predictive value, such as CD200 for diagnosis of Chronic Lymphocytic Leukemia (CLL). It is specific except nodal MCL – Mantle Cell Lymphoma (37). PPV can be very useful when a combination of monoclonal antibody percentage positivity, fluorescence density, and percentage of cells in a cell population is used. Scoring for Myelodysplastic Syndrome is a good example for this approach (38). Even though sensitivity is low for both “Ogata” and “Red” scores, when combined their high specificity and positive predictive value make these scoring systems a useful tool for clinical diagnosis. Note: The lysis methods can interfere in the results.
Negative predictive value	Test negativity in healthy, true negative fraction, ability of a test to identify the absence of disease at a particular decision threshold. Note: The negative predictive value varies with the prevalence of the disease in the population tested.	The following question is addressed: How likely is non-disease given the test results? The negative predictive value (NPV) describes the perspective of a physician or a patient in view of a negative test result: It is the probability that the patient has not got the disease (as defined by an independent reference standard) given a negative test result (post-test probability). The NPV depends on the prevalence of the disease. Its value corresponds to the clinical situation where the test is applied. When a test has a NPV > (100%-prevalence) it might have a good diagnostic performance (taking into account a similar consideration for the PPV in parallel).	The presence or lack of an antigen provide information on Negative Predictive Value (NPV). A good example is 100% NPV (prevalence = 4%, PPV = 5.4%) for neutrophil expression of CD64 for excluding sepsis cited by (39): 100 patients with suspected sepsis were investigated and authors found an excellent negative predictive value for CD64 (100% sensitivity and 100% NPV), although specificity was low in this study (28% specificity). CD34 counts for bone marrow transplantations, depending on the absolute counts, and percentage, also have a PPV and NPV for success of the transplantation. Another example for NPV is the use of specific CD4+ T cell responses to discriminate the latent and active tuberculosis cases. NPV is as high as 92.4% (prevalence = 19.1%, PPV = 80%) for this approach (40).
Likelihood ratio	“Likelihood ratio” means the likelihood of a given result arising in an individual with the target clinical condition or physiological state compared to the likelihood of the same result arising in an individual without that clinical condition or physiological state (1). For a binary test the positive and negative likelihood ration are determined. The positive diagnostic likelihood (DLR+) ratio is the probability of a positive test result given the disease divided by the probability given the non-disease. DLR–: Test negativity in healthy, true negative fraction, ability of a test to identify the absence of disease at a particular decision threshold.	DLR+: The following question is addressed: By how much does the test change knowledge of the disease status? In other words, the positive diagnostic likelihood ratio describes directly the gain in information a test provides (whereas the PPV can only be interpreted when it is set into relationship with the prevalence). Formally, the DLR+ is the ratio of post-test odds and pre-test odds of the disease given a positive test result. Practically, it is calculated as sens/(1-spec) [in case of a binary test]. Meaningful tests should have DLR+ > 1. DLR–: The following question is addressed: By how much does the test change knowledge of disease status? In other words, the negative diagnostic likelihood ratio describes directly the gain in information a test provides (whereas the NPV can only be interpreted when it is set into relationship with (100%-prevalence)). Formally, the DLR– is the ratio of post-test odds and pre-test odds of the non-disease given a negative test result. Practically, it is calculated as (1-sens)/spec [in case of a binary test]. Meaningful tests should have DLR– <1.	Sometimes presence or absence of one marker effect the likelihood ratio of flow cytometry results as CD49d for CLL prognosis. CD49d is an unfavorable prognostic marker, comparison of likelihood ratio along with other performance measures indicated that omission of CD49d significantly reduces the prognostic power of the prediction models (41). Efforts for development of better analysis and interpretation software in cytometry systems are ongoing. Use of Z-scoring in classification of cells expressing multiple fluorophores, use of spillover in actively scoring events, and the successful classification of multiple fluorophores using a single detector within a flow cytometer is suggested by Lawrence et al. (42) There are too many factors for determination of positive (DLR+) and negative likelihood ratio (DLR–) in cytometry based clinical use. Clinical status of patient, stage of disease, accuracy of the test, environmental and genetic factors, age, gender, accompanying diseases all effect the likelihood ratio. An example for this complicated situation is bronchoalveolar lavage fluid immunophenotyping for CD4+/CD8+ cells in diagnosis and follow up of pulmonary sarcoidosis. A meta-analysis performed for determination of likelihood ratio found PLR as 4.04 while NLR was 0.36 (Likelihood ratios >30 and <0.33 are considered as strong indicators to rule in or rule out a diagnosis, respectively). This suggest that immunophenotyping of CD4+/CD8+ has low ability to discriminate sarcoidosis from non-sarcoidosis (43).

## Parameters for Validation

Analytical parameters for a specific assay must be determined independently in each laboratory that performs the assay. This should include, if applicable, analytical sensitivity and specificity, trueness (bias), precision, repeatability, intermediate precision, reproducibility, accuracy (resulting from trueness and precision), limits of detection, limit of quantitation, measuring range, linearity, cut-off, determination of appropriate criteria for specimen collection and handling, control of known relevant endogenous and exogeneous interference (cross-reactions), and robustness. Definitions and specifics for FCM are given in Table 3. Analytical performance characteristics given by EU-IVD-R that shall be stated by manufacturers to state “fitness for purpose” need to be maintained during the lifetime of an IVD. As commented in this table, although it should be noted that not all performance characteristics can be validated for every flow cytometric setting. And, finally, even if it would be feasible, the full method validation for each modified or novel analysis, each sample type, and each pathological issue would be outrageously expensive and time-consuming. For transparency reasons, we recommend to document which characteristics were not validated and the underlying reasons.

**Table 3:** Analytical performance characteristics given by EU-IVD-R that shall be stated by manufacturers to state “fitness for purpose” need to be maintained during the lifetime of an IVD.

**Term**	**Definition/explanation**	**Comments**	**Specific considerations for flow cytometry**
**ANALYTICAL PERFORMANCE**			
Analytical sensitivity	Quotient of the change in an indication of a measuring system and the corresponding change in a value of a quantity being measured (Slope of an empirical calibration curve (indirect reference measurements).	There are several definitions of “analytical sensitivity” with different meanings. Within this document we use the term “analytical sensitivity” to describe any performance evaluation in terms of LoB, LoD (see below) and/or LoQ (see below), as in the IMDRF framework. Another general term, which is used by CLSI (20), is “detection capability.” The term is not used in the CLSI evaluation protocols. It is recommended to refer to LoB, LoD, LoQ (see below).	Sensitivity refers to the precision and accuracy of rare events and dim antigen measurements. It is important for measurable/minimal residual disease analysis for leukemia, lymphoma, and multiple myeloma samples. For this type of samples, to reach to high level of sensitivity, minimal number of cell counts are important. Lower Limit of Detection (LOD) is the lowest number of cells counted. Usually 10–50 events are enough for adequate calculations. At least 50 events are necessary for lower limit of quantitation (LOQ). LOD and LOQ can be obtained by below formula: LOD or LOQ = (MRD Cluster/total cells acquired) ×100% (44). Calibration of flow cytometer is not considered here because this must follow manufacturers advise.
Analytical specificity	Note: analytical specificity resembles the concept named selectivity. Selectivity gives an indication of how strongly the result is affected by other components in the sample (45). The CLSI EP07 (46) uses this term.		Specificity is how well a flow cytometry test determines the specific cell population and/or the antigen evaluated. This includes all stages of cytometry analysis from sample collection to patient report release. Sample type, antibody selections, panel design, analysis, standardized interpretation of results are important for the analytical specificity (23). Heterotypic antibodies and cross-reactivities as well as uncommon target epitopes can cause aberrant results. Specificity of antibodies cannot be verified but should be given by providers, preferentially as CE-labeled IVD.
Trueness (bias)	Closeness of agreement between the average of an infinite number of replicate measured quantity values and a reference quantity value (8).	Measurement trueness is inversely related to systematic measurement error. The estimate for the systematic error is the bias. The bias is measured as the difference between an average of quantity values and a reference quantity value used as measure for “true quantity.”	Not required/not possible to establish in majority of immune-oncological applications. There is no gold standard. Therefore, most EQA use consensus values.
Precision	Closeness of agreement between indications or measured quantity values obtained by replicate measurements on the same or similar objects under specified conditions.	Comment: Measurement precision is usually expressed numerically by measures of imprecision, such as standard deviation, variance, or coefficient of variation under the specified conditions of measurement. Precision is inversely related to the random error of a measurement and covers several reasons of it. Thus, the precision is measured by evaluating its components (repeatability, intermediate precision and reproducibility). These components refer to specific conditions under which the experiments are performed. Thus, the definition of the conditions is essential for understanding the related precision component.	Intra-assay and inter-assay precision need to be assessed. Intra-assay precision is determined when same sample is measured repeatedly under the same conditions, and how close the results are. Accepted criteria for immunophenotyping are co-efficient variation (CV) of 10–25% (31). For rare events and dimly staining antigens higher CV values may be accepted. Inter-assay precision (reproducibility) is measured by obtaining the variability between the instruments, analysts, and different laboratories.
Repeatability	Measurement precision under a set of repeatability conditions of measurement with *repeatability* condition: condition of measurement, out of a set of conditions that includes the same measurement procedure, same operators, same measuring system, same operating conditions and same location, and replicate measurements on the same or similar objects over a short period of time	The most effective and sufficient experiment follows a hierarchical design. Within this design, several variance components (e.g., repeatability, operator-to-operator-variability and day-to-day variability) are evaluated together. A hierarchical design with nested factors (e. g., 3 operators investigate on 5 days 3 replicates (3 × 5 × 3 measurements). In case of 1 factor and repeatability, the analysis can be performed using simple Excel-Spreadsheets.	Repeatability can be measured by preparing 3–6 samples in at least three replicates. In one run all samples can be tested. This assay should be run on one instrument by one technical person. It should be measured on the most representative type of samples and the most representative cell subset, at different levels. Within the statistical analysis the results per sample are pooled. This analysis, however, requires the homogeneity of the results over the concentration range.
Intermediate precision	Measurement precision under a set of intermediate precision conditions of measurement with *intermediate precision* condition: condition of measurement, out of a set of conditions that includes the same measurement procedure, same location, and replicate measurements on the same or similar objects over an extended period, but may include other conditions involving changes		This type of measurement can only be assessed with QC samples when available. Because of the sample shortage and the cost of the analysis, repeats cannot be done as many times as usually recommended in biochemistry. Dorn-Beineke et al. recommend higher numbers (17, 18). We believe that 11 repeats (47) would be safer as long as the sample volume makes it possible. We recommend hierarchical designs. Supplement II shows the example of an experiment investigating 1 factor together with repeatability.
Reproducibility	Measurement precision under reproducibility conditions of measurement with reproducibility condition: condition of measurement, out of a set of conditions that includes different locations, operators, measuring systems, and replicate measurements on the same or similar objects		Reproducibility measurements for instruments can be performed by two different technicians (one for each instrument). If there is an inconsistency between the results, then the technical person and the instrument need to be evaluated. Stabilized IQC if available can be analyzed daily, keeping in mind that the stabilization procedure alters cell shape and marker expression. Again, because of the sample limited volume and the cost of the analysis, we propose testing at least one IQC per level, per type of sample available, per operating day. Inter operator reproducibility can be estimated by comparing IQC analyses between different operators on different times. We recommend hierarchical designs. Supplement II shows the example of an experiment investigating 1 factor together with repeatability.
Accuracy (resulting from trueness and precision),	Closeness of agreement between a measured quantity value and a true quantity value of a measurand.	Accuracy is a conceptual term describing the agreement of a single measured value with the true quantity. Inaccurate measured values could be caused by systematic (bias)= and random (imprecision) errors. The “true quantity” is an ideal state. Accuracy is therefore not directly validated but is covered by validation of trueness and precision. *Systema*tic error: Component of measurement error that in replicate measurements remains constant or varies in predictable manner (7). *Ran*dom error: Component of measurement error that in replicate measurements varies in an unpredictable manner (7). A random error shows up when a measurement is repeated under the same conditions.	If bias could not be established, accuracy given by precision. Comparison of results from different laboratories may be used for calculation of accuracy. Participation to external QC/proficiency testing programs when available will provide the most useful information for systematic error. Systematic error = Mean of bias (48). Random error = Standard deviation of bias
Limits of detection	Measured quantity value, obtained by a given measurement procedure, for which the probability of falsely claiming the absence of a component in a material is β, given a probability α of falsely claiming its presence.	The LoD signals the presence of a measurand in the sample. Lowest measured quantity value at which it is statistically shown that “something” of the component is in the sample (qualitative statement). α and β are typically set to 5%.	MRD is a good example. There are different options for detection of LOD. FMO (fluorescence minus one) can be used as LOD tool, by omitting the antibody of interest. Using healthy donor samples is also possible. Rare results require high cell counts to be analyzed (Poisson challenge). Cell identification is based on a good separation of positive/negative labeling and the sensitivity of detection that is limited if the fluorescence of the conjugate is poor or if the antigen is expressed at low density on cells, e.g., below 1,000 molecules/cell (49). Antigen density can be quantitatively measured using FCM and reference values have been published by the European Working Group on Clinical Cell Analysis (49–51). As an example, B cell antigens have density varying from 12 ± 2 CD21 antigens per cells, 27 ± 3 CD19 up to 149 ± 29 CD20 (49).
Limit of quantitation	Lowest amount of measurand in a sample can be quantifiably determined with stated acceptable precision and trueness under stated experimental conditions		Similar tools used for obtaining LOD can be used for LOQ determination. Spiking leukemia samples with known dilutions into healthy donor samples can also provide data for determination of LOQ. This resolution allows to distinguish two populations in a mixture of particles that differ in mean signal intensity (52). It must be adapted to the medical need by adapting the number of total events to be acquired. For the lymphocyte count, a 10–50 cell/μL (10^−3^ of leukocytes) resolution is usually enough while high sensitivity detection, below 0.10–1 cell/μL require an acquisition of at least 10^−4^ to 10^−5^ of leukocytes) or even less (10^−6^ to 10^−7^) for the assessment of minimal residual diseases.
Measuring range	Working interval set of values of quantities of the same kind that can be measured by a given measuring instrument or measuring system with specified instrumental measurement uncertainty, under defined conditions.		For fit for purpose validation, verification with a minimum of ten donors are recommended when validated IVD/CE assays are used (46). This is not the case for rapidly alternating tests in immune oncology. Purified subsets and depleted matrix close to the sample characteristics (e.g., whole blood) are not available for proper spiking tests. This should be repeated for each of the several subsets analyzed in one analysis. We propose that the linearity of the analysis can be approached, on ONE representative cell subset, by spiking a sample with high concentration of the subset (e.g., Lymphoproliferative syndrome) in one sample with a lymphopenia in the considered subset, as low as possible (e.g., patient treated with depleting biotherapy such as anti CD20 monoclonal antibody). We recommend performing 6 to 10 serial dilutions (1/3 or 1/4) of a sample with a subset at concentration from 10^4^ to 10^5^ cell/μL, in a sample with same subset at concentration <10 cell/μL as much as possible. Usual sensitivity for reliable routine T cell count requires an acquisition of at least 10,000 leukocytes.
Linearity	Assuming no constant bias, the ability (within a given range) to provide results that are directly proportional to the concentration (amount) of the measurand in the test sample.	According CLSI EP06 (19), the data are analyzed by linear, quadratic and cubic regression. If one of the quadratic or/and cubic regression parameters are significant, the deviation from linear model has to be checked whether they are relevant or not (by regarding them in view of the repeatability of the measurements)	Linearity can be achieved by use of standard calibrators to control the efficacy of fluorescence detectors on the measurement device. To achieve linearity measurement on biological samples can be possible by spiking healthy donor samples with known cells such as leukemia cells.
Cut-off	The cut-off refers to a specific measurement value which is used as a decision limit to distinguish between different categories of test results, typically between positive and negative test results.	Cut-off level is a test value or statistic that marks the upper (or lower) boundary between diagnostic categories, i.e., between negative (acceptable or unaffected) results and positive (unacceptable or affected) results (53).	Cut-off values are used for clinical performance determination and for qualitative tests as detection of allergen-specific basophil granulocytes. For quantitative analysis (expression strength), the minimal level of fluorescent intensity measured on each cell is directly dependent on (a) the antigen density (42, 49), (b) the optimal immuno-labeling (54) and (c) the fluorochrome properties. The use of calibration beads (55, 56) allows to check instrument performance over time and to provide direct comparison of data between different instruments (57, 58).
Determination of appropriate criteria for specimen collection and handling		Common criteria are defined in the pre-analytical handbook of laboratories.	For different matrix (bone marrow, peripheral blood, body fluids) and different analysis (such as platelets or activated platelets), appropriate specimen collection and handling instructions should be validated and be provided in written format. Clotting, contamination, or mucous must be avoided.
Robustness	Show, that specific factors have no influence on measurement results	When the aim is to show no influence of the factor, the analysis with equivalence tests (TOST) is appropriate. To use criteria like “no statistical significance (*p* value >0.05)” as found with a conventional *t*-test are not correct from statistical point of view since imprecise measurements would lead to false negative results, whereas precise measurements could lead to significant but not relevant deviations and therefore to false positive results.	Robustness can be measured by measuring the tested parameters' impact on results.

## Performance Targets (Table 4)

For a validation, we must define acceptance criteria in advance as part of the validation plan. Performance targets must enable the reviewer of the validation data to state whether the determined performance capability is adequate for the intended use or not. In some cases, the assessment may lead to the conclusion that further investigation is necessary or that restrictions exist for the analytical procedure that need to be considered in routine diagnostics.

**Table 4:** Specific method validation and acceptance limits.

	**Method specificities**		**Type of analyses**			**acceptance limits**
**Validation**	**Dates, operators**	**Quantitative**	**High-sensitive**	**Qualitative**	**Functional**	
Risks	Sample, reagents operator, data analysis	+	+	+	+	
Sample type	Typical cite other accepted	+	+	+	+	
Repeatability	RSD (%)	11 repeats 2 levels. preferentially combined with reproducibility in a hierarchical precision experiment (Supplement II)	+	NA	7–10	<10%
Reproducibility	IQC Levey-Jennings, eventual interlaboratory comparison	18-24 tests 2 levels bias to mean of labs preferentially combined with repeatability in a hierarchical precision experiment (Supplement II)	NA	NA	NA	<10–15% Precision index <2* repeatability
Trueness (bias)	EQC usual workflow	3–5/year 2 levels	+	NA	NA	<15%
Global uncertainty	Uncertainty^2^ = Precision^2^ + Accuracy^2^/ √3	+	+	NA	NA	
Working range linearity	6–10 × 1/3 or 1/4 dil. At least one subset 1 test, 1 sample type	clinical relevance e.g., 5–5,000 cell/μL, generic form	+	NA	+	Set deviations from linearity in relationship with repeatability
LOQ (low)	% of leukocytes Event acquired	10^−3^ % (10 cell/μL) 2–5 × 10^4^ events	10^−4^ −10^−5^% for 10^5^-10^6^	Extrapolated		
Sample stability	10 fresh samples on 2-3 days	Subpopulations labeling MFI	+	+	+	<10%
Stability of pre-mixed reagents	2–3 fresh samples fresh/old mix 2 IQC one mix on time	Subpopulations (%) labeling MFI	+	+	+	<10%
Interferences	Atypical phenotype “alert gates”	Generic form	+	Extrapolated	Extrapolated	
Carry-over	3 (very) high, 3 low, 3–5 times	(L1-L3)/(meanH-L3) generic form	+	Extrapolated	Extrapolated	<1%
Method comparison	At least 30 double tests mean difference, slope	Multiple instruments change of technique	Few tests	–	–	Difference~0, Slope~1 95% CI within +/– 10.15%
Reference values	30 healthy donors (F/M) initially, to be verified by data from daily routine >100 healthy donors	Most representative Parametric analysis: Two sided: mean +/–2 SD, One sided: mean + or – 1.645 SD, presentation with 90%-confidence intervals non-parametric analysis: percentiles	–	–	–	
Special groups	literature	Children, elderly.	–	–	–	

There are only few international recommendations for tolerated variability in flow cytometric diagnostics. As a rare example, references are proposed in Westgard data base for CD4+ T cells counts although no technical conditions are defined such as system used, internal standards, or even units that are critical in Quality Assurance of the technique as discussed before (15, 59).

## Experimental Set-Up

The design of validation experiments must follow general rules but can be adapted if necessary. Especially, very often the small number of samples, the limited time in which the samples can be processed, and the small volume accessible are limiting factors. The best options to overcome this are multi-sample or multi-center approaches. The aspect of sample size as an important part of experimental design is mentioned below.

## Statistics for Validation Experiments

There have been strong efforts to improve the quality of statistical approaches in design and analysis of method validation experiments in the last years. There are four principle features of statistical methodology which should be considered (Box 3).

Box 3 **Four principle features of statistical methodology**

**Figure 1:**
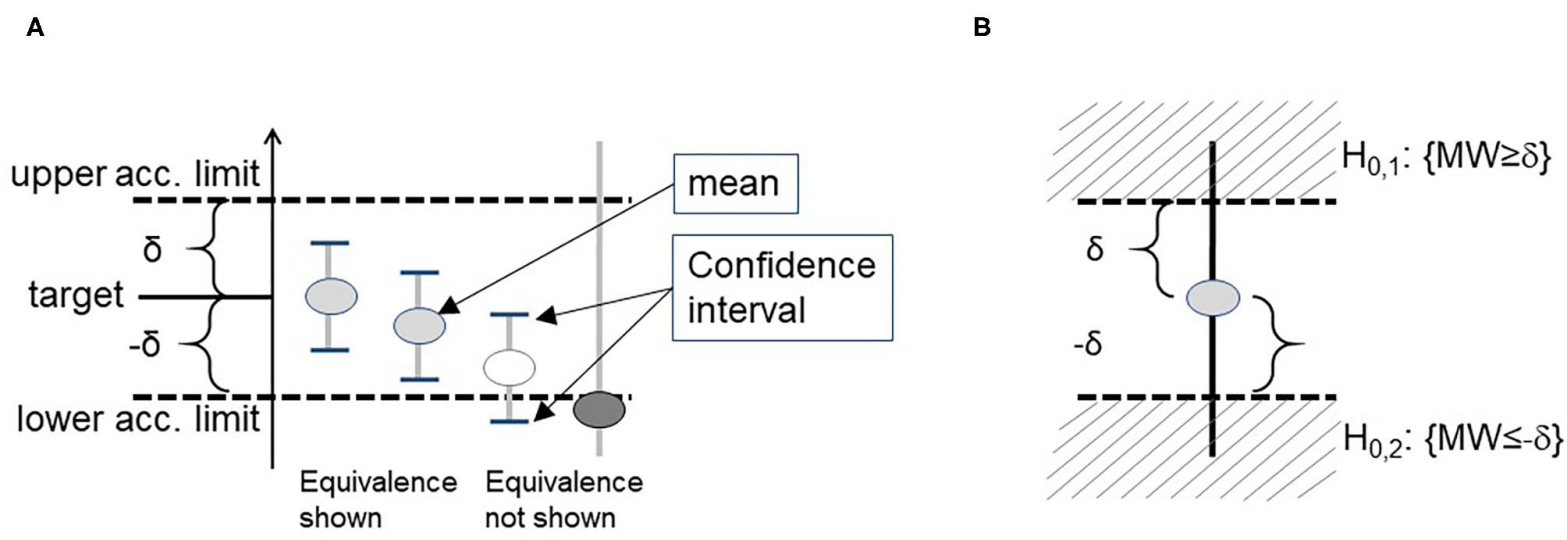
Demonstration of a statistically proof using confidence intervals **(A)**. When this problem is formulated as a statistical test, it refers to the two 1-sided test approach (TOST) **(B)**.

**Figure 2:**
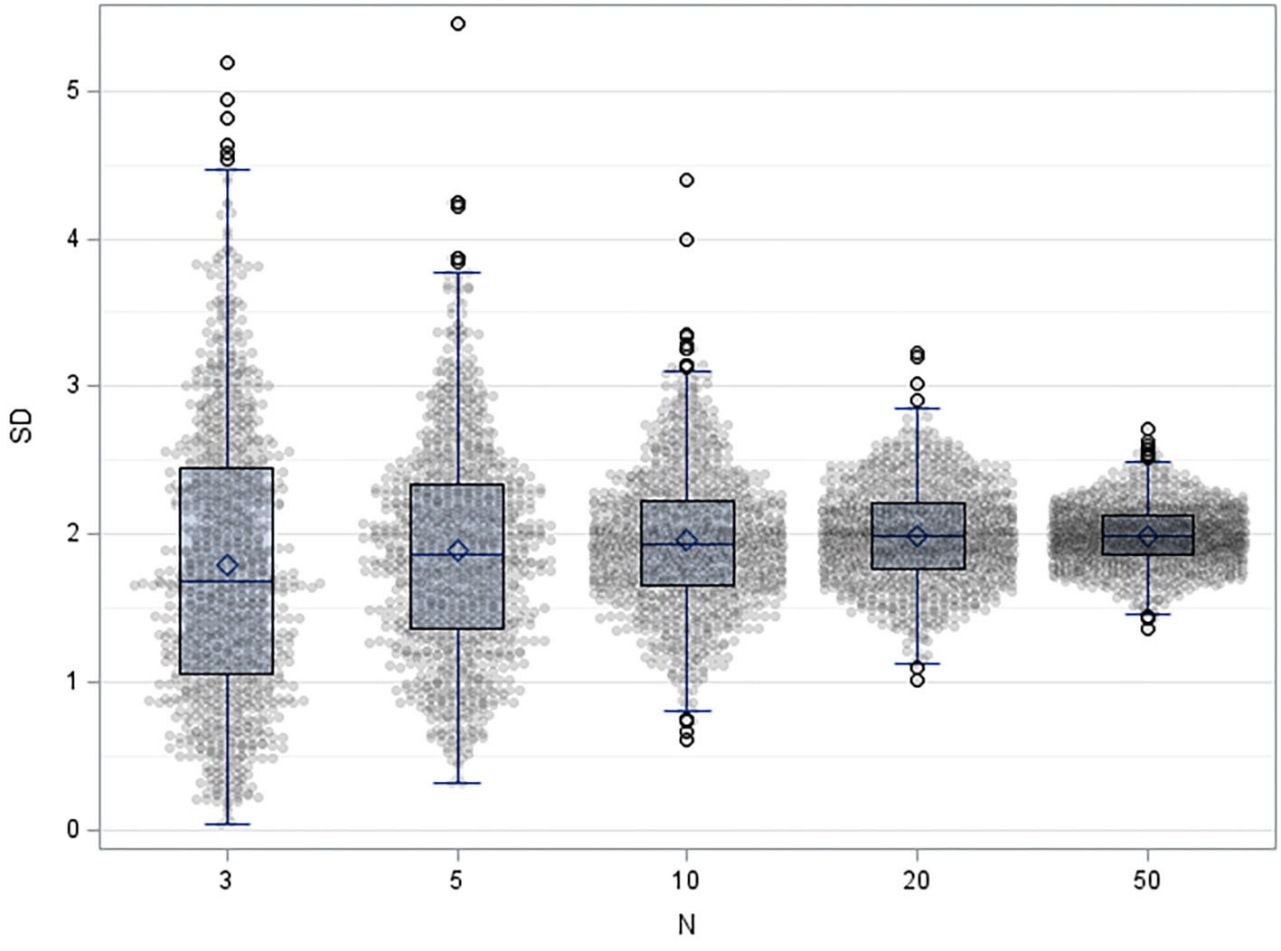
Result of 1,000 simulation of results of repeatability experiment when 3, 5, 10, 20, and 50 replicates are used, with mean=10 and standard deviation =2, shown as dot-plots with overlying Box-whisker plots.

1) Stringent use of prospectively defined acceptance criteria, which are used as limits in later statistical tests.
2) Any result (statistical term: estimate) should be reported together with its uncertainty, typically expressed as a confidence interval (CI). Within the framework of statistical analyses, the location of the CI is considered in comparison to the acceptance criteria. If the confidence interval does not overlap with the acceptance limits, the validity is proven. It should be noted that conclusions can only be drawn in this direction: if an acceptance criterion is within confidence interval, no conclusion is possible.
3) We therefore recommend the application of equivalence tests: often, the aim is to show a difference of zero, e.g., in experiments evaluating robustness or selectivity, where the results of distorted measurements should be equivalent to results of an undistorted control experiment. After establishing acceptance criteria prospectively, the CI of the difference of distorted and undistorted measurement results should be within acceptance criteria around zero (Figure 1). The related statistical test is the TOST approach (two one sided *t*-tests, see Supplement III for details) (60).
4) Finally, sample sizes should be determined by power calculations. Statistical tests differ in their robustness to small numbers of cases. The user should know and estimate the behavior of the algorithms used. Procedures that are more reliable for small case numbers should be preferred. An example is given for robustness in Table 5. The sample sizes required for sufficient test power should be known before validation. The resulting test power should be included in the evaluation, especially if the sample size is smaller. Practically, the sample size is determined using software, formulas, statistically derived recommendations as CLSI-guidelines (19– 21) and tabulations (see Table 5 for TOST in this paper). We cannot recommend oversimplified so-called practical approaches (“ <5 replicates were found adequate to validate assay imprecision levels below the 5–10% CV” (61). Here, simulations (62) performed on common spreadsheet software or R could be helpful, Figure 2 shows such considerations for uncertainty of standard deviations one could achieve in simple repeat experiments when 3, 5, 10, 20, and 50 replicates are used.

**Table 5:** Sample sizes necessary to demonstrate equivalence via TOST in a paired design when acceptance criteria cover the range (−1, 1), in dependence on standard deviation of the pairwise differences, real deviation, and power.

**Sample sizes N for acceptance criteria (-1, 1)**		**Real deviation**						
		**0**	**0.1**	**0.2**	**0.25**	**0.3**	**0.4**	**0.5**
**StdDev**	**Power**	***N***						
0.25	80%	4	4	4	4	4	4	4
0.5		4	4	5	5	5	6	8
0.75		7	7	8	8	9	12	16
1		11	11	12	13	15	19	27
1.25		15	16	18	19	22	29	41
1.5		21	22	25	27	30	41	58
1.75		28	29	33	36	41	54	78
2		36	37	42	47	53	71	101
0.25	90%	4	4	4	4	4	4	4
0.5		5	5	6	6	7	8	11
0.75		8	9	10	11	12	15	21
1		13	13	15	17	19	26	36
1.25		19	20	23	26	29	39	55
1.5		26	28	32	36	41	55	79
1.75		35	37	43	49	55	75	107
2		45	48	56	63	72	97	139

*Overall alpha level is set to 5%. The proportional relationship between acceptance criteria, standard deviation and real deviation can directly be used to derive samples size for other scenarios. Example: Acceptance criteria: +/– 30%, CV of the differences = 15%, real deviation = 0%, power = 80% → sample size = 4 (achieved by using StdDev = 0.5, deviation = 0 and power = 80%). The CV of differences should be the precision of the single experiment multiplied with 1.4 (= square root of 2)*.

In addition to statistical methodology for analysis of validation experiments, the following practical aspects of analysis should be discussed:

- Deviation from normal distribution: Statistical tests determining deviations from normal distribution are not useful for demonstrating a lack of normal distribution. One can apply visual inspection of histograms (no outliers, symmetrical gauss-shaped distribution, or QQ-plot presenting a straight line). Moreover, one can use the fact, that replicates of a measurement are very often normally distributed. Finally, a transformation of data could be useful (see below).
- Outliers or better “aberrant values”: Statistical methods could help to identify whether an aberrant value is an outlier, however, the decision whether the outlier has to be incorporated in the data is not a statistical task, since an imperfection of the method, e.g., to handle matrix effects, could be the reason. Rules how to handle outliers must be defined in advance. An easy way to enlighten the situation is to perform the measurements in duplicates and in a random order: when both replicates are aberrant values although they were processed on different positions in the work flow, they cannot regarded as outliers but to be real values. When only one of the replicates is aberrant, it might be an outlier which can be handled according the internal SOP how to handle outliers.
- Counting data like single cells or particles, especially in the low range (1 … ~ 20) follow the Poisson distribution. This distribution has some specific properties in that large imprecision is just given by the distribution and cannot be improved by experimental efforts. It is out of the scope of this report to address the specific approaches necessary for Poisson-distributed data, see (63–65) for further reading. Note that square root transformation of count data is helpful within statistical analysis (66) in the same sense as log-transformation is often applied.
- In case of low sample sizes one can statistically average (other term: pool) the results over the samples. An example are precision analyses: If only a small number of replicates are available per sample, a pooled precision can be calculated as the square root of the sum of squared standard deviations (or by specific methods related to variance components). We refer also to the next chapter, §4, and to Supplement II). However, homogeneity of the variances (standard deviations do not systematically depend on concentration) is a prerequisite for the pooling and—if not given—could be achieved by appropriate transformation of data (ln, square root).
- ln-transformation: In case of natural log (ln)-transformation, the standard deviations obtained for ln-transformed data can directly be read as CV in the originally scaled data (for instance: SD=0.1 in ln-transformed data CV=10%in originally scaled data, valid up to 30% CV).

## Evaluation of the Results

Validation is successful when the acceptance criteria are met. If these performance criteria are not met, this may be for the following reasons: (1) the estimated target value is outside of the criteria, (2) uncertainty of the target value is too high and does not allow a decision, or (3) representative samples are absent in the experiment (e.g., missing positive specimen). Whereas, in case 1 the method itself must be modified, in both latter cases, an extension of the validation process can be indicated. A common approach is a two-step clearance procedure with an extended sample collection phase that increases the sample size by continuously evaluating the results of measured patient samples and accompanying data on quality assurance. In such cases, the completion of the validation process should be declared preliminary and clear instructions should be given on the measures still to be taken. The reservations resulting from a preliminary clearance status should be formulated and reported to the customers.

## Our Proposal for the Introduction of Laboratory Developed Tests in Accredited Laboratories

Considering all difficulties in the accreditation process of FCM analysis and all discussions in dedicated meetings, we propose a reasonable and pragmatic solution (Table 4). We also include the consideration that the majority of samples with pathological phenotypes are rare or only available in small volumes and cannot be tested too many times for repeatability and reproducibility.

1. New antibodies are often only available in research-only vials. They are not always labeled with the desired fluorochrome. To check the specific binding, it has proven to be best to use two different or differently labeled antibodies in the validation phase. In addition, Full Fluorescence Minus One control (FMO) must be used to ensure that there is no spill-over into other channels.
2. The reagent quality is guaranteed by the manufacturer, but some alteration can appear during the delivery from the provider to the laboratory according to the conditions. The basic requirement is a stable measuring instrument, which is ensured by daily checking with fluorescent beads. Furthermore, fluorescence intensity of novel antibody batched should be checked with antibody binding standard beads. It would be a huge endeavor to check each single vial before doing analysis but daily checks of the fluorescence intensity of control blood is a good way to validate not only the reagent quality but also the labeling process and the state of the sample. The proper labeling can be easily checked by using a pre-recorded template where each cell populations should fit into the gates positioned at the usual place. So, it is critical to validate each analysis with checking all dot plots graphs.
3. The premix stability must be compared to freshly mixed antibodies on a fresh sample or following IQC. Because labeling intensity may gradually decrease with time, not only population phenotypes but also median fluorescence intensity should be compared.
4. Cells are analyzed from different sample types. The analyses are similar to each other within prespecified acceptance criteria regarding the sample type excepting some minor adaptations for the sample preparation. We recommend doing the method validation on one of the most representative type of samples such as peripheral blood or bone marrow aspirate. Sample types which are explicitly unsuitable for the considered test, but which may arrive in the laboratory should be specified and the reasons leading to the rejection of the order should be described.
5. Several cell subsets are analyzed in one analysis (one analysis, several results). However, each subset cannot be fully tested individually. As all subsets are exposed to the same preparation and same risks of errors, we propose to consider that the performances observed for two representative subsets and one type of sample can be used as reference for Quality Assurance for the analysis of the other cell subsets and sample types. The selected sample type should correspond to the most frequently occurring ones. Subsets chosen should be of clinical relevance. Expected values should cover a wide measurement range or at least include both low and high measurement signals.
6. The effect of transportation and storage on sample stability must be tested typically on 10 samples for the acceptable storage duration (2–3 days, dependent on target cells). Again, TOST approaches are helpful for the analysis: the mean of deviations due to a possible instability should be within predefined limits around zero. Modern approaches include using a regression analysis and setting the confidence band of the regression line into relationship with prespecified acceptance criteria (67).
7. Carry-over can be evaluated by measuring consecutively 3 times the sample with the highest content (e.g., Lymphoproliferative disorder) and 3 times the sample with the lowest content (e.g., depleted sample in biotherapy) the day they are both available. The high values should be at least 100 times higher than the lower content. As the risk does not depend on the subset identification, it can be extrapolated to all other subsets. perform the experiment in at least 3 cycles and use non-inferiority testing (= one sided equivalence test) for statistical analysis (68).
8. **Bias estimation/method comparison:** When two or more instruments are used independently or as backup in case of instrument malfunction, assays should be performed repeatedly on both machines for comparison. In clinical FCM, number of repeats is often limited by the number of samples required for valid results, therefore alternative procedures must be found. Statisticians commonly recommend performing at least 30 assays on both systems and the CLSI EP 9 guidance (69) recommends using 40 samples for the laboratory and 100 samples for the manufacturer. When the TOST is used for analysis of difference plots (Supplement III), sample sizes provided in table 5 can be used. For analysis Bland-Altman plots (70, 71) as well as specific regression methods like Passing-Bablok regression (72) or Deming regression are recommended (73). Note that simple ordinal linear regression as well as the correlation coefficient *r*^2^–although often used—are not appropriate (74, 75). Especially the *r*^2^ does not detect proportional and constant biases, e.g., one could achieve a *r*^2^=1 even when one method measures the double of the other method. For analysis the TOST or similar approaches are helpful. In the Bland-Altman plot the CI of the mean of sample-wise differences should be within predefined limits around zero. When regression methods are applied, the CI of the slope should be within predefined limits around 1 and the intercept within predefined limits around zero, or the CI of biases calculated from the regression line vs. line of equality at specific concentrations (typically 3 values within the measurement range) should be within predefined limits.
9. **Precision:** The most effective way to estimate several components of variability follows a hierarchical design with nested factors (e.g., 3 operators investigate on 5 days 5 replicates (3 × 5 × 5 measurements) (21). Within this design, several variance components (e.g., repeatability, operator-to-operator-variability, and day-to-day variability) are evaluated together (Supplement II). Especially repeatability is pooled over several experimental units. In case of one parameter and repeatability, the analysis can be performed using simple spreadsheet-software like MS Excel. It is also possible to pool the results over several samples and use fewer replicates within the factors, however, homogeneity of variances must be achieved for the analysis then, eg. by transformation of the measurement values (ln, square root). One should note that the CI-approach (which would use the one-sided upper confidence limit here) is not common in precision evaluations in the laboratory medicine community. It was shown that the level of variability was mainly related to the size of the population. Accordingly, Tosato et al. (76) described a CV of 2% for large T cell populations, 5.5% for B cells, and 12.5% for NK cells in 10 independent measurements of an IQC for clearly defined markers (Immuno-Trol Cell Control; Beckman Coulter).
10. In the absence of any international standard to validate EQA samples, accuracy can often be approached only by inter-laboratory comparisons in EQA. The targeted accuracy (EQC bias) should be below 15%.
11. Calculation of measurement uncertainty combines reproducibility and accuracy. Because of the rarity of EQA, we propose to use IQC for this calculation. When investigating measurement uncertainty, it must be considered that the various cytometric stains used are not independent variables. This influences the propagation of errors in a positive way (25).
12. As discussed, the determination of the complete working range is not possible. We propose that the linearity of the analysis can be approached, on ONE representative cell subset, by spiking a representative cell line into one sample with a low count in the considered subset. We recommend performing 10 serial dilutions. The usual sensitivity for reliable routine T cell count requires an acquisition of at least 10 000 leukocytes.
13. Definition of limit of quantitation (LoQ) must be adapted to the medical need by adapting the number of total events to be acquired. For the lymphocyte count, a 10–50 cell/μL (10e-3 of leukocytes) resolution is usually enough while high sensitivity detection, below 0.10-1 cell/μL require an acquisition of at least 10e-4 to 10e-5 of leukocytes) or even less (10e-6 to 10e-7) for the assessment of minimal residual disease.
14. **Robustness, specificity:** When measurements of distorted and not-distorted samples must be compared, it is the aim to show a missing difference. As introduced and explained above, the TOST can be used to show the equivalence. Depending on the design, paired or unpaired measurements must be regarded, whereby a paired design is more powerful. Beside other software, free of cost MS Excel-tools are available (https://www.acomed-statistik.de/en-gb/statistical-tools-download.html#TOST). The sample size depends on width of interval included by acceptance criteria, the expected real difference and its standard deviation as well as on the assumed α (typically 5% and β errors (typically 10–20%). The following Table 5 provides sample sizes for a paired design (all samples are measured under both conditions; the difference of both results is evaluated in analysis). Supplement III provides an example.
15. Reference ranges can be preliminarily calculated from 31 to 35 assays, however CLSI guideline EP28 (77) recommends 120 to 135 healthy donors. The CLSI recommendation refers to a non-parametric estimation of percentiles. Lower sample sizes require the application of complex parametric methods (78). As the reliability of reference ranges is limited if the proposed sample size used, the 90% confidence interval of both lower and upper reference interval limits should be calculated and critically reviewed (10, 11). By doing this, an inappropriate sample size becomes obvious. Even in case of recommended sample sizes the CI are surprisingly wide. More accurate determination specific to the population to be tested (e.g., babies/children, elderly over age 75, or gender) cannot be measured in each lab for practical, economical, and ethical reasons and can be taken from international data available although they are rarely standardized (79–83). Here, quantile regression for age groups is superior but not realistic for most laboratories. A simplified proposal has been described by Özcürümez et al. (84). For complex phenotypes, subset identification regarding antibody combination and gating strategy must be clearly described in the SOP. Gating strategy must be double-checked repeatedly. A simple tool is the control of the quality of the sample in FSC/SSC plots and each single labeling vs. SSC that gives information on the quality and specificity of the immunostaining (85–88).
16. As accreditation is a continuous process, we propose method validation should be repeated periodically. If established, an IQC program should be done every operating day. Precision, working range, and contamination should be checked repeatedly every 1 or 2 years. Normal ranges should be verified every 10 years.

## Document Hierarchy

All method descriptions and characteristics must be reported in detail and continuously updated in the accreditation records, SOP, and LIMS. These reports must be easy to read and in a fixed layout.

Because of protocol flexibility and frequent evolution in FCM, details on the method description must be frequently updated. Typical examples would be:

- Removing or replacing an antibody or one clone or
- Adding a washing and red blood cell lysing step, if incomplete lysis was occasionally observed in some samples.

If the same information is cited at different positions along the accreditation forms or in the LIMS, there is a very high risk for discordance. Redundancy severely impairs readability and makes document maintenance risky and error-prone and consequently should be avoided as much as possible.

Lots of facts are common to several assays, e.g., environment, the instrument characteristics, the method principle, procedures on standardization, sample preparation, samples/reagents management, security, and risks. Results of different sub-populations are frequently complementary subsets of some parent populations. Several combinations of antibodies (panels) can have common features. As an example, a panel for diagnosis of leukemia can require 6–8 assays with a common backbone. Multiple results are produced and should then be considered together for interpretation. An accreditation report must combine multiple results (one analysis—several results) or possibly multiple assays as a panel (several analyses—one result), in the same file and preferentially lists of information are presented in a table for readability.

**Figure 3:**
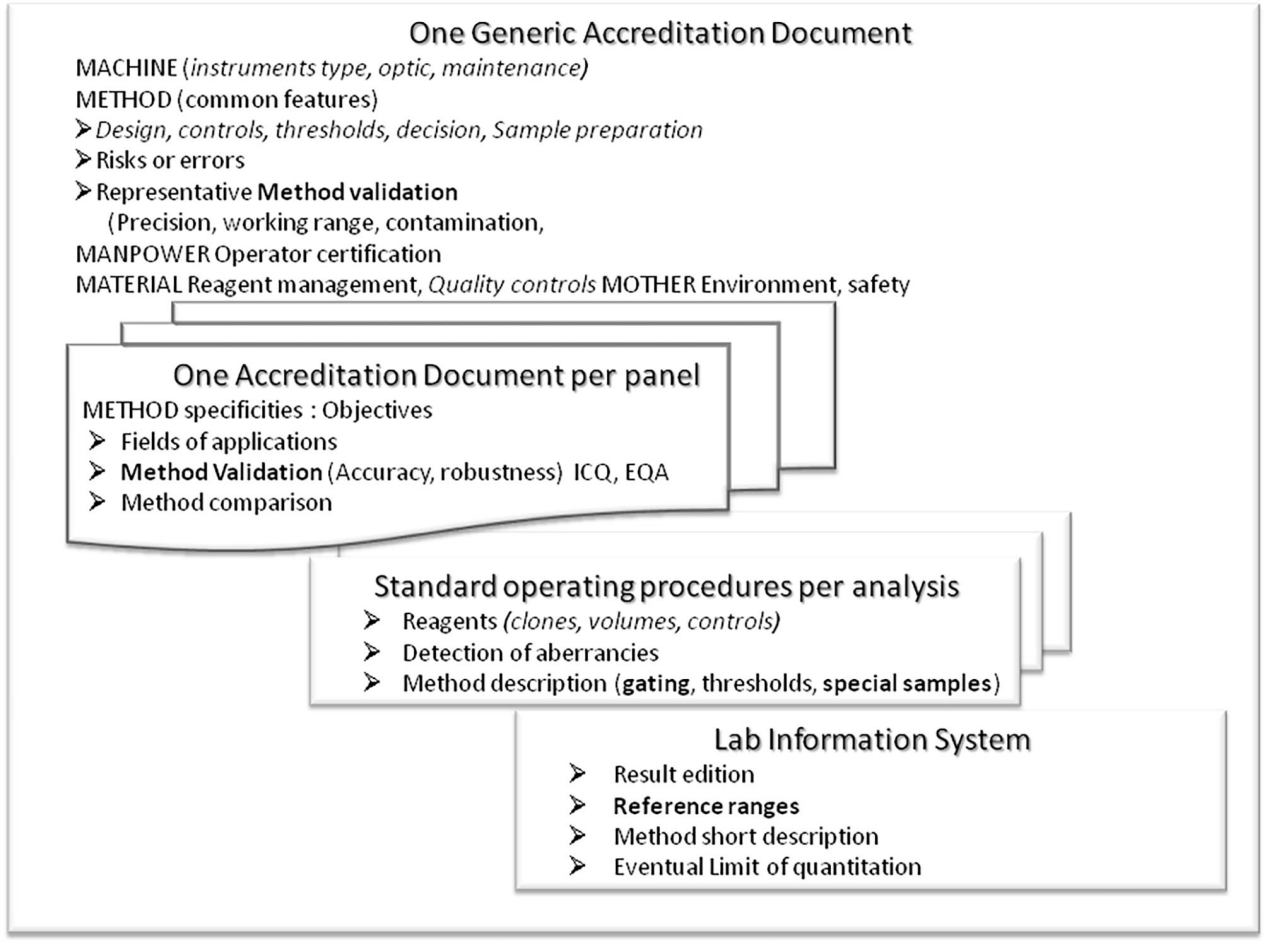
Presentation of the structure proposed for the accreditation documents. A generic form is to record and report all common information (including environment, material, management, manpower) and method characteristics that cannot be tested for each panel. Then specific forms should be written individually per panel (several parameters, several assays). Technical details (antibodies, clones, conjugates, gating strategy, risks of error, and guidelines for interpretation) should be presented in an easy-to-update SOP. Results with technical and reference information should be managed by the laboratory informatics system to be published for correct interpretation. Any redundancy should be avoided for safety and management reasons.

For efficacy and safety reasons, we propose organizing the documents on 4 different levels (Figure 3):

1. Any common information must be gathered (“factorized”) in a common “generic” accreditation form as much as possible.
2. The specificities (reagents, method, performances) must be detailed in analysis-specific forms: One analysis “one analysis—several results” or “several analyses—one result” in one common accreditation form
3. The technical specificities required for daily practice at the bench and interpretation (gating strategy, reagents specificities, etc.) must be specified in the analysis-specific SOP.
4. The information necessary for interpretation and a report with the results (reference values, LoQ, units, etc.) must be collected in the LIMS.

The generic description must mention all common critical points; operators and supervisors (education, training, CPD/CME, information), environment (storing requirement; work space ergonomics, hygiene, air quality, humidity, room temperature), measurement principles, material management (reagents, standards and samples; conditioning, storing, transportation, label/identity, acceptability/rejection, registration, tracking); instrument characteristics including cytometer and accessory instruments, optical bench, instructions, daily checks for fluidic and optical stability, principles for settings, spectral overlap compensations, standardization of signal detection, check-up, maintenance. Some common components of method validation can also be gathered in this generic form such as sample preparation including process for immuno-labeling, washes, red blood cell lysis, fixation, storing, calibration, absolute counting strategy; units, standards, data acquisition, interpretation; reference to peer recommendations (ICSH), quality control management, risks of error, result validation, recording, transfer, and reporting. Part of the method characteristics is also common. Risks of Error (RoE, caused by pipetting errors of antibodies or internal standards, incomplete lysis of red blood cells, clots, centrifugation, cell loss), and effects on fluorochromes (between fluorochromes, energy transfer, steric hindrance, matrix effects such as bile salts or antibodies to fluorochromes), their detection (minimal count of cells, correct cell location in dot plots) and their prevention and correction must be listed. Most RoE are common to all FCM analyses and thus should be detailed in the generic form rather than in the panel-specific information. Lists of technical parameters/materials (antibodies, fluorescence dyes, clones, provider, concentration) must be presented in tables that are easier to read instead of text and attachments.

The analysis-specific records must include the specificities for the environmental conditions and method (lysis, washing steps, internal standards, dyes, templates, expected normal, and aberrant populations) and should be conceived according to clinical relevance (awareness for doublets or dead cells relevant, relevance of percentages of absolute values, delta check, limit of detection). If required, these forms can also merge data from different analyses like non-stimulated and stimulated cells or different panels for the distribution of T cell clonotypes. These analyses are usually closely related, sharing many features (sample type, incubation steps, lysis, washing buffers, centrifuge, incubation). Each detail that can be changed or adapted frequently should not be included here like reagent lots, pipetting, volumes respective cell numbers of cells, additional washing steps, rare sample types), but in the SOP. These specific forms (per analysis) should also contain as much as possible information on analysis characteristics. Some assays validation could be approached from a related analysis (working range, linearity, limit of quantitation) that cannot be done for all analysis but can be extrapolated from other analyses and described in the generic form (like absolute count linearity, limits of detection, or contamination. This is also true for common errors (like pipetting, reagents quality, centrifugation, red blood cell lysis, cell separation procedures, washing).

The SOP must detail all technical specificities, the method principles, specific reagents (references, isotypes, clones, providers, fluorochromes, and conjugated antibodies), concentrations (based on titration or manufacturer recommendations), calibration, specific requirements on sample preparation, acquisition parameters (delay, number of events to acquire), and expiration date. As phenotype definition is critical, each subset should be clearly described (antibody, gating strategy, population hierarchy) and be referred to peer literature when available. FSC/SSC plots provide valuable information on the sample quality and debris. Doublets and dead cells must be excluded from analysis. This is easily done for dead cells because a live/dead staining such as 7-Aminoactinomycin D or aggregation of dead cells helps to exclude them. Doublet exclusion can be done by gating scatter height vs. area. Population overlap (e.g., lymphocytes and monocytes) must be avoided by gating strategies such as Boolean gates. Backgating and use of color codes are good tools to check the quality of the gating. The template with typical results including dot plots, level of fluorescence intensity expected, and most common and atypical types (sub-populations) should be described. It is recommended that the template include “alert gates” for unexpected combinations to provide a signal in case of improbable phenotypes.

LIMS should include all information needed to interpret the results. Subset definitions, LoQ, reference values must be listed in the data management system (LIMS).

As discussed, operator competence in FCM directly relates to quality assurance. Different projects supporting education and certification at an international standard are under development by various international societies: ESCCA, ICCS, or ISAC. The educational sessions (courses, congresses, etc.) visited by staff members should be clearly described and competence should be tested. All documents must be archived.

## Educational Sources

FCM technique is rarely formally taught in general biological fields and even less in diagnosis. Only a few countries grant certificates or have study programs in this specific technique like the French University Certificate on Cytometry. The International Society on Analytical Cytometry (ISAC) proposes an internationally recognized qualification in basic cytometry (International Cytometry Certification Exam (http://cytometrycertification.org/) with continuous follow up. The International Federation of Clinical Chemistry and Laboratory Medicine (IFCC) offers courses and schools, organized by the working group flow cytometry WG-FC (http://www.ifcc.org/). The European Society for Clinical Cell Analysis (ESCCA) promotes continuous education and training in annual international schools and courses as well as professional development and evaluation on specific topics. In 2017, ESCCA has initiated an examination for their members to become an ESCCA-certified cytometrist. ESCCA European cytometry certification includes two levels of certification, one for cytometry operators and one for cytometry specialists (http://www.escca.eu).

## Conclusion

We propose a “generic” accreditation method for all common steps (instrument settings, protocol design, and data analysis and decision strategy), a detailed description of each method (protocol, RoE), and quantitative validation of a few representative methods. More detailed and frequently updated data such as reagent characteristics, gating strategy, typical results, and reference data must be described in the SOP and, in part, also in the LIMS. The flow cytometry technique is entering a mature state with better-defined methodology for instrument settings, protocol design, standardization, and data analysis and interpretation. Nonetheless, because of its large scope and flexibility and for economic reasons, FCM accreditation procedures must be pragmatic, feasible, and efficient. Our proposal also defines several premises for further harmonization of the processes connected with the validation of FCM assays. In a next step, for instance, the community of laboratories that frequently perform such validation routines could now compile a collection of sample records and may develop “best practice” templates for the evaluation of validation data.

## Data Availability Statement

All datasets generated for this study are included in the article/Supplementary Material.

## Author Contributions

CL, GY, TK, FP, KP, MS, MÖ, and US wrote parts of this manuscript, double-checked the submitted draft, and agree to be accountable for the content of the work.

## Conflict of Interest

TK is the owner of Acomed Statistik. The remaining authors declare that the research was conducted in the absence of any commercial or financial relationships that could be construed as a potential conflict of interest.

## Abbreviations

CAR-T: Chimeric antigen receptor transduced T lymphocytes
CE: Conformité Européenne
CI: Confidence interval
CLSI: Clinical and Laboratory Standards Institute
CME: continuing medical education
CPD: continuing professional development
CV: coefficient of variation
DLR: diagnostic likelihood ratio
EQA: external quality assessment
EQC: external quality control
ESCCA: European Society for Clinical Cell Analysis
EU-IVD-R: European Regulation on *in vitro* diagnostic medical devices
FCM: flow cytometry
FMO: fluorescence minus one
FSC: forward scatter
ICCS: International Clinical Cytometry Society
ICSH: International Committee for Standardization of Hematology
IMDRF: International medical device regulators forum
IQC: internal quality control
ISAC: International Society for Advancement of Cytometry
IVD: *in vitro* diagnostic medical devices
LDT: laboratory developed tests
LIMS: laboratory information management systems
LoB: limit of blank
LoD: limits of detection
LoQ: limit of quantification
MRD: minimal residual disease
QQ-plot: quantile-quantile-plot
RoE: risks of error
SD: standard deviation
SOP: standard operating procedure
SSC: side scatter
TOST: test of one-sided significance
VIM: International vocabulary of metrology.
