# Supplementary material for: Flow Cytometric Analyses of Lymphocyte Markers in Immune Oncology: A Comprehensive Guidance for Validation Practice According to Laws and Standards
Source: Front Immunol. 2020 Sep 17;11:2169. doi: 10.3389/fimmu.2020.02169 (PMC7528430; doi:10.3389/fimmu.2020.02169)
Supplement: Supplementary file 1 [file Data_Sheet_1.pdf]

Supplement I

Introduction

- Objective of the technique, general description, fields of applications, risk analysis

Manpower

- Qualification, training, continuous education
- Sample preparation; instrument use
- Technical validation, data transfer, biological validation
- Quality assurance
- Material management

Measurement

- Pipetting

Mother Nature, environment conditions

- Instruments, reagents, samples, temperature, humidity, dust, light
- Working conditions, ergonomics
- Security, hygiene

Material

- Samples and quality controls:
    - Collection, transport, registration
    - Validation, pre-analytical storing
    - Conditioning, preparation for analysis, post analytical storing, discard
- Standards and Reagents:
    - Ordering, reception, registration,
    - Tracking, validation, storing, stock management,
    - Manufacturer information, alerts, vigilance

Machine

- Flow cytometer, sample processor, computer
- Description, instruction for use, maintenance, breakdown

Method

- Description
    - Measurement, units, standards, reference values
- Setting
    - Standard operating procedures management, storing, updating
    - Principles for sample preparation: washing, lysis, pipetting, fixation, additives
    - Principles for protocol design, settings, compensation, standardization
    - Quality controls management
- Risk management
    - Type of samples, conditions of acceptance
    - Environment
    - Operator training
    - Tracking
- Characteristics
    - Working range; limit of detection/quantification;

Risks of errors

- Contamination risk
- Interferences
- Reagent/signal detection stability
