# Supplementary material for: Flow Cytometric Analyses of Lymphocyte Markers in Immune Oncology: A Comprehensive Guidance for Validation Practice According to Laws and Standards
Source: Front Immunol. 2020 Sep 17;11:2169. doi: 10.3389/fimmu.2020.02169 (PMC7528430; doi:10.3389/fimmu.2020.02169)
Supplement: Supplementary file 2 [file Data_Sheet_2.pdf]

Supplement II – Example of precision analysis

1 Data

The data are from an experiment investigating repeatability and variance of a factor (e. g. operator).

The data refer to a 3 (operators) x 5 (replicates) design using 3 samples.

Sample	factor	repl 1	repl 2	repl 3	repl 4	repl 5
1	1	9.9	9.8	11.7	11.9	9.7
1	2	9.6	9	8.9	8.9	8.5
1	3	11.9	11.3	12.9	11.4	12
2	1	18.7	20	22.2	17	23.4
2	2	16.4	16.4	16.6	18.3	17.7
2	3	22.0	23.6	23.7	25.2	22.8
3	1	46.2	51.4	52.4	51.9	57.4
3	2	41.7	48.7	49.2	43.7	44.2
3	3	61.8	58	64.1	58.8	62.2

Tab. 1: data

2 Visualization and simple description

The descriptive statistics are provided by sample.

Sample	Mean	StdDev	%CV
1	10.49	1.43	13.6%
2	20.27	3.13	15.4%
3	52.78	7.26	13.8%

Tab. 2: simple descriptive statistics

The description shows dependency of standard deviations on concentration, the relative standard deviation (%CV), however, does not depend on concentration. In that case, natural-log-transformed values (ln) can be used for analysis. The advantage of using ln-transformed values is that the standard deviation (e. g. 0.1) obtained for these transformed values can directly be read as %CV for original scaled values (e. g. 10%). This approximation holds for all %CVs <30%.

It is always recommended to visualize the data.

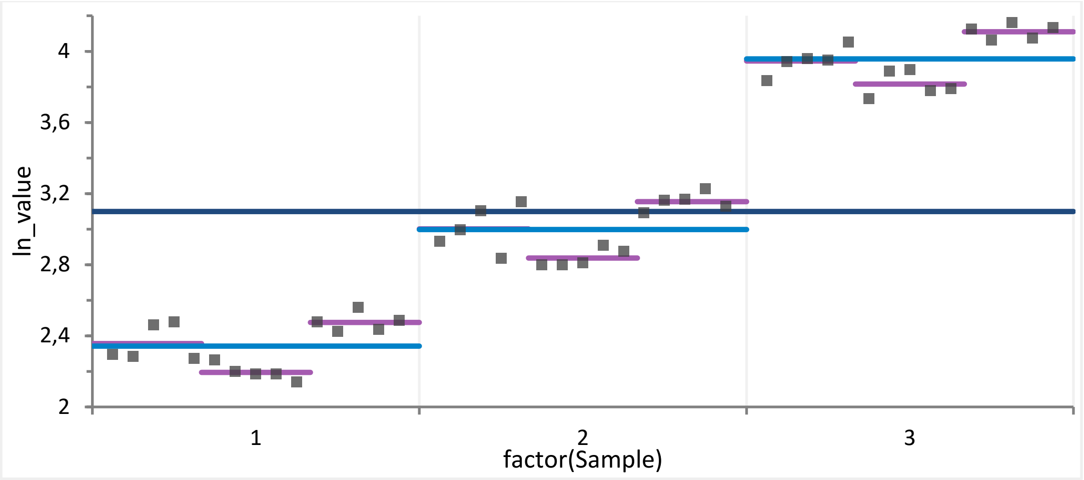

Fig. 1: Variability-plot of data Visualization of ln transformed values by sample (numbers on x-axis) and factor. The horizontal lines show the means overall (dark blue), over sample (blue) and by factor (pink).

Homogeneity of variances is given for ln-transformed values can be concluded. Thus, the results for precision obtained per sample can be pooled. Note, that the pooling of standard deviations por %CVs is NOT performed by calculation of the their mean, but by the square root of the mean of squared values, see below.

3 Estimation of variance components by sample, based on spreadsheet-software

ANOVA tables by sample are calculated.

Applying MS Excel on ln transformed data arranged as in Tab. 1, using Data – Data Analysis – ANOVA: single factor, one obtains the following menu:

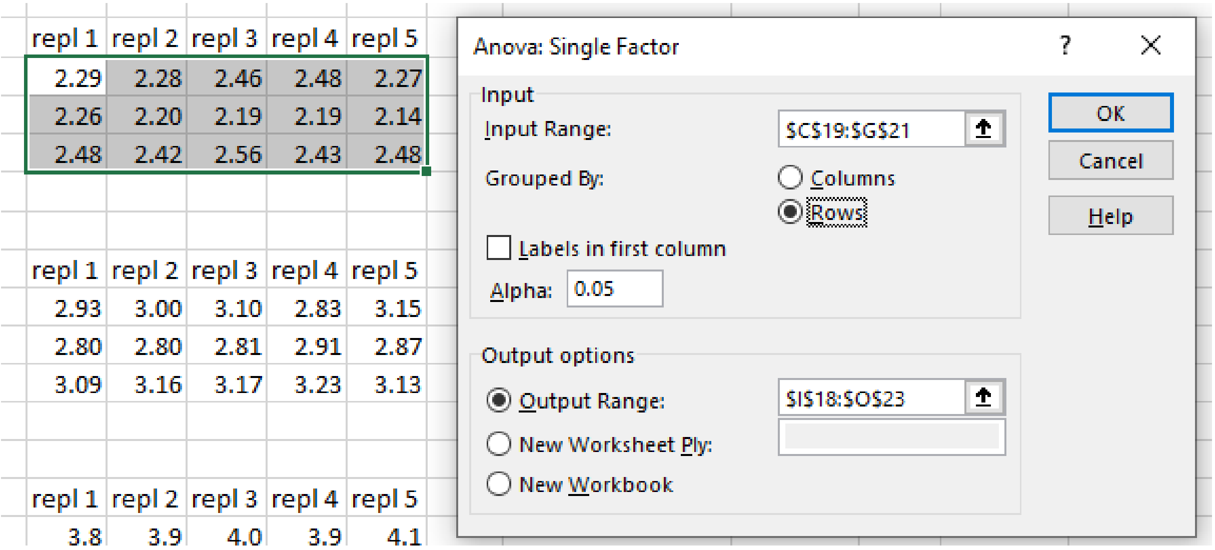

Fig. 2: Screenshot: how to obtain an ANOVA table via MS Excel (data → data analysis → Anova: Single Factor)

Following ANOVA tables are calculated.

repl 1	repl 2	repl 3	repl 4	repl 5		ANOVA			
2.29	2.28	2.46	2.48	2.27		Source of Variation	SS	df	MS
2.26	2.20	2.19	2.19	2.14		Between Groups	0.19925	2	0.099625
2.48	2.42	2.56	2.43	2.48		Within Groups	0.060471	12	0.005039
						Total	0.259721	14	

repl 1	repl 2	repl 3	repl 4	repl 5		ANOVA			
2.93	3.00	3.10	2.83	3.15	2.93	*Source of Variation*	*SS*	*df*	*MS*
2.80	2.80	2.81	2.91	2.87	2.80	Between Groups	0.251977	2	0.125989
3.09	3.16	3.17	3.23	3.13	3.09	Within Groups	0.086606	12	0.007217
						Total	0.338583	14	

repl 1	repl 2	repl 3	repl 4	repl 5		ANOVA			
3.8	3.9	4.0	3.9	4.1		*Source of Variation*	*SS*	*df*	*MS*
3.7	3.9	3.9	3.8	3.8		Between Groups	0.21734	2	0.10867
4.1	4.1	4.2	4.1	4.1		Within Groups	0.051496	12	0.004291
						Total	0.268836	14	

Tab. 3: ANOVA tables (MS Excel): "within-group" refers to "within factor" → repeatability, "between-group" refers to "between factor", only relevant parts of the ANOVA tables are shown.

Following formulas are used to calculate the variances (index w – within group, b – between group) from the ANOVA table. The standard deviations are calculated as the square root.

Within-factor variance (Repeatability): ${\sigma_w}^2 = MS_w$

Between-factor variance: ${\sigma_b}^2$ =max$\left[0, \frac{MS_b - {\sigma_w}^2}{N}\right] = max\left[0, \frac{MS_b - MS_w}{N}\right]$

with : N – number of replicates

Total: ${\sigma_t}^2 = {\sigma_w}^2 + {\sigma_b}^2$

Thus, following results are obtained_

Sample 1:

Repeatability: $\sqrt{0.005039}$ =0.071. This Standard deviation for ln transformed values can read as %CV for originally scaled values: %CV_{rep} = 7.1%

Between-factor standard deviation: $\sqrt{(0.099625 - 0.005039)/5}$ =0.138, %$CV_{betw\text{-}factor}$=13.8%

Total: $\sqrt{0.071^2 + 0.138^2}$ = 0.158, %CV_{total}=15.8%

Sample 2:

Repeatability, sample 2: $\sqrt{0.007217}$ =0.085, %CV_{rep}=8.5%

Between-factor standard deviation: $\sqrt{(0.125989 - 0.007217)/5}$ =0.154, %$CV_{betw\text{-}factor}$=15.4%

Total: $\sqrt{0.085^2 + 0.154^2}$ = 0.176, %CV_{total}=17.6%

Sample 3:

Repeatability, sample 3: $\sqrt{0.004291}$ =0.066, %CV_{rep}=6.6%

Between-factor standard deviation: $\sqrt{(0.10867 - 0.004291)/5}$ =0.144, %$CV_{betw\text{-}factor}$=14.4%

Total: $\sqrt{0.066^2 + 0.144^2}$ = 0.159, %CV_{total}=15.9%

Given the homogeneity of variances, one can pool the results by taken the square root of the mean of squared standard deviations.

Overall repeatability: $\sqrt{\frac{0.071^2 + 0.085^2 + 0.066^2}{3}}$ = 0.074, %CV_{rep} = 7.4%

Overall between-factor standard deviation: … %$CV_{betw\text{-}factor}$ = 14.6%

Overall total standard deviation: … %CV_{total} = 16.3%

4 Estimation of variance components by sample, based on statistical software

The advantage of using statistical software is the availability of confidence intervals. Moreover, the analysis of precision experiments with 2 or more factors is possible, and unbalanced data (datasets with missing values) can be analysed.

The disadvantage, however, is, that typically a huge number of options is available. The results between different software as well as between versions of the same software might slightly differ which makes it difficult to validate the analyses. It is recommended to gain experience with known data sets before using such software. The software version to be used should be prospectively defined in the experimental protocol.

4.1 Preparation of data for analysis

For statistical analysis with statistical software (R, Analyse-It® or other), the data should be provided in long format.

sample	factor	replicate	value	ln_value
1	1	1	9.9	2.29
1	1	2	9.8	2.28
1	1	3	11.7	2.46
1	1	4	11.9	2.48
1	1	5	9.7	2.27
2	1	1	18.7	2.93
2	1	2	20	3.00
2	1	3	22.2	3.10
2	1	4	17	2.83
2	1	5	23.4	3.15
3	1	1	46.2	3.83
3	1	2	51.4	3.94
3	1	3	52.4	3.96
3	1	4	51.9	3.95
3	1	5	57.4	4.05
1	2	1	9.6	2.26
1	2	2	9	2.20
…	…	…	…	…

Tab. 4. Data in long format

4.2 Analysis with software R

The analysis with software R requires programming skills in this software.

Within R, one should install package VCA, which was developed for analysis of hierarchical precision experiments according CLSI guideline EP05-A3.

The following code can be used (for pooled analysis, ignore the results for total and sample):

```
#load Package VCA
library(VCA)
path<-file.choose(new=FALSE)
study<-read.csv(path, header=TRUE, sep=",", dec=".")
str(study)
fit<-anovaVCA(ln_value~sample/factor,study)
inf <- VCAinference(fit, VarVC=TRUE)
```

```
as.matrix(inf, what="SD", digits=6)
```

Note: The code for precision analysis is just 1 line (fit…). The both last lines refer to estimation of CI. Note that results for CI could differ between software packages. Note that other methods for estimation of variance components are available (e. g. REML [`remlVCA`]- restricted maximum likelihood instead of ANOVA, which is advantageous in case of missing values).

Whereas the package VCA (see https://cran.r-project.org/web/packages/VCA/index.html for details) was especially programmed for analysis of precision experiments, one might use other packages which are programmed for the underlying statistical models (so called mixed models).

```
#load Package lme4
library(lme4)
…
res<-lmer(ln_value ~ (1|factor),data=study, REML=T)
VarCorr(res)
confint(res)
```

4.3 Analysis using Software Analyse-It®

The results obtained with software Analyse-It (a commercial Excel-Add-On, www.analyze-it.com) are presented.

Sample 1

Component	SD	Exact / MLS 95% CI
Within factor	0.071	0.051 to 0.117
Between factor	0.138	0.065 to 0.886
Total	0.155	0.096 to 0.889

Sample 2

Component	SD	Exact / MLS 95% CI
Within factor	0.085	0.061 to 0.140
Between factor	0.154	0.072 to 0.997
Total	0.176	0.111 to 1.001

Sample 3

Component	SD	Exact / MLS 95% CI
Within factor	0.066	0.047 to 0.108
Between factor	0.144	0.070 to 0.926
Total	0.159	0.096 to 0.928

Tab. 5: Results (for ln-transformed values) obtained with software Analyse-It®. From the standard deviations (SD), the %CV can be achieved by multiply it with 100% (valid until CV~30%).

The confidence intervals for between-factor results are really large, so these results underlie a large uncertainty. This is due to the relative small sample size for the experiment per sample (15 measurements).

Using the sample as a naïve factor in a 2-factor analysis, one achieves the **pooled results** with smaller confidence intervals.

Component	SD	Exact / MLS 95% CI	
Within factor	**0.074**	0.060	to 0.096
Between factor	**0.146**	0.090	to 0.327
Within Sample	**0.163**	0.117	to 0.335

Tab. 6: Results pooled over samples (for ln-transformed values) obtained with software Analyse-It®. From the standard deviations (SD), the %CV can be achieved by multiply it with 100% (valid until CV~30%).

5 Discussion

A simple 1-factor experiment can be analysed using spreadsheet-software like MS-Excel.

However, the calculation of uncertainty of the results as expressed by 95%-confidence intervals requires statistical software. Given the homogeneity of the variances, the pooling of the results over the samples improves the reliability, however, the sample size chosen in the example (3 samples x 3 factor-item x 5 replicates) is still small. Using more samples, factor items or replicates might be appropriate to achieve larger sample sizes. In the CLSI-guideline EP05-A3 about 75 .. 80 samples are used for an hierarchical experiment with 2 factors, thus a 5 samples x 3 factor-item x 5 replicates -experiment (→ 75 samples) is more efficient.
