# Supplementary material for: Flow Cytometric Analyses of Lymphocyte Markers in Immune Oncology: A Comprehensive Guidance for Validation Practice According to Laws and Standards
Source: Front Immunol. 2020 Sep 17;11:2169. doi: 10.3389/fimmu.2020.02169 (PMC7528430; doi:10.3389/fimmu.2020.02169)
Supplement: Supplementary file 3 [file Data_Sheet_3.pdf]

Supplement III – Example of a robustness analysis with equivalence test

1 Sample size

A robustness experiment is planned for evaluating the bias introduced by a factor in comparison to the undistorted experiment. Each sample will be measured with both methods.

The acceptance criteria have been set to ±15%.

The repeatability of the method is 10%. The related %CV of the differences is $\sqrt{2} \cdot 10\% = 14\%$

We assume complete robustness, so the bias should be 0. To be sure, we assume that bias is 5%.

We use a power of 80% (probability that robustness is shown when the experiment is robust) and an alpha level of 5% (probability that we show robustness when the experiment is not robust).

These values are used for a sample size calculation, we use table 5 of the paper for this purpose. We recalculate all parameters for limits ±1 (so divide all values by 15%). So the StdDev =14%/15% ~ 1, the real deviation is 0.33. The table gives us values for real deviation of 0.3 (N=15) and 0.4 (N=19), so we interpolate to N=17.

So we should use a sample size of 17.

2 Data

From the experiment we get the following data. The table presents the calculated differences by sample, too.

Sample	Undistorted measurement	Distorted measurement	Difference	%Difference
1	12.1	16.6	4.5	31.5%
2	15	16.9	1.9	13.3%
3	13.9	15.1	1.2	8.4%
4	14.5	15.4	0.9	6.3%
5	13	17.1	4.1	28.7%
6	13.5	13.8	0.3	2.1%
7	14	15.4	1.4	9.8%
8	15.7	16.9	1.2	8.4%
9	15.9	12.6	-3.3	-23.1%
10	12.7	15.1	2.4	16.8%
11	16.4	14.1	-2.3	-16.1%
12	14.2	14.5	0.3	2.1%
13	16.6	11.9	-4.7	-32.9%
14	15.8	14.5	-1.3	-9.1%
15	13.4	14.2	0.8	5.6%
16	11.7	15.6	3.9	27.3%
17	15.7	14.7	-1	-7.0%

Tab. 1: data with calculated difference (distorted – undistorted) and %difference=difference/mean(undistorted)

3 Visualization and spreadsheet software analysis

The following table provides the analysis as performed with MS Excel, whereby Standard formalus for mean (AVERAGE[RANGE], standard deviation (STDEV.S[RANGE] and width of confidence interval (from limit to mean) (CONFIDENCE.T(0.1;STDEV[RANGE];COUNT[RANGE) have been used.

Note that the 90% confidence interval is calculated which is related to the 5% alpha level which is used for both single tests used in the TOST, which both must be significant to show equivalence. So, simply said, the 90%-two-sided confidence-interval is the intersection of 2 one sided 95%-confidence intervals.

	Undistorted measurement	Distorted measurement	Difference	in %
Mean	14.36	14.96	0.61	4.2%
StdDev	1.51	1.45	2.54	17.7%
N			17	17
width 90%-CI			1.07	7.5%
Mean - CI			-0.47	-3.3%
Mean + CI			1.68	11.7%

Tab. 2: Analysis with MS Excel.

A mean %difference =4.9% (90%-CI: -3.3% .. 11.7%) related to undistorted mean results.

Since the confidence interval is within acceptance criteria ±15%, robustness of the method within the acceptance criteria can be concluded.

4 Analysis with TOST

4.1 Analysis with software Analyse-It®

Using the values for % difference, one obtains the following results for 90% CI as well as for TOST:

Location

Mean	0.0424
90% CI	-0.0328 0.1176
SE	0.0431

TOST (two-one-sided t-tests)

Equivalence interval	-0.1500 0.1500

H0	t statistic	DF	p-value
μ ≤ -0.15	4.47	16	0.0002
μ ≥ 0.15	-2.50	16	0.0119

Max p-value	0.0119[1]

H0: μ ≤ -0.15 or μ ≥ +0.15
The mean of the population is not within the equivalence interval.
H1: -0.15 < μ < +0.15
The mean of the population is equivalent to 0 within the equivalence bounds (-0.15, +0.15).
[1] Reject the null hypothesis in favor of the alternative at the 5% significance level.

Fig. 1: Result for TOST obtained with software Analyse-It®

4.2 *Analysis with a free available Excel-tool*

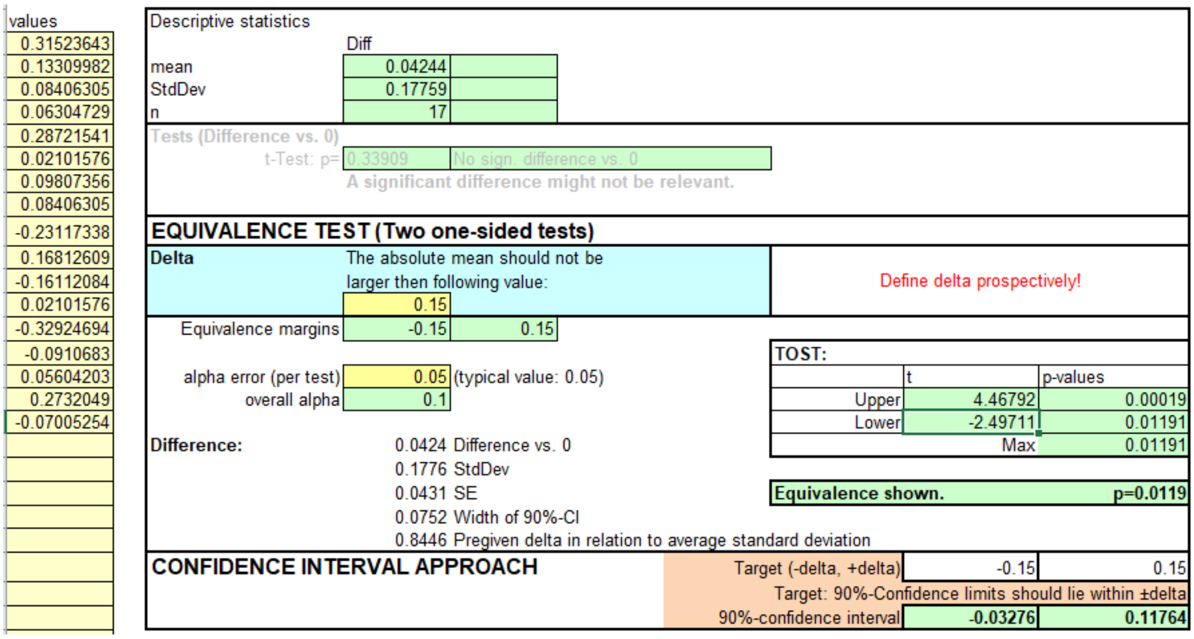

Fig. 2: Result for TOST obtained with free available Excel-tool ((https://www.acomed-statistik.de/en-gb/statistical-tools-download.html#TOST)

4.3 *Analysis in R*

```
c<-c(0.315236427,
     0.133099825,
     0.084063047,
     0.063047285,
     0.287215412,
     0.021015762,
     0.098073555,
     0.084063047,
     -0.23117338,
     0.168126095,
     -0.161120841,
     0.021015762,
     -0.329246935,
     -0.091068301,
     0.056042032,
     0.273204904,
     -0.070052539
)

library(equivalence)
tost(c, epsilon = 0.15, conf.level = 0.95)
```

Fig. 3: Code used within R

The following figure shows the result. Interestingly, the confidence interval is named “95%-two one-sided confidence interval (TOST-interval)” to avoid confusion in terms of the width of the confidence interval and the alpha level.

```
data:  c
df = 16
sample estimates:
mean of x
0.0424436

Epsilon: 0.15
95 percent two one-sided confidence interval (TOST interval):
 -0.03275564  0.11764283
Null hypothesis of statistical difference is: rejected
TOST p-value: 0.01190606
```

Fig. 4: *Result for TOST obtained with R*

5 Discussion

The TOST analysis can be obtained with spreadsheet software as well as with statistical software packages. Interestingly, this feature is relative new and might therefore not have been used in older literature.
